# Revisiting the TALE repeat

**DOI:** 10.1007/s13238-014-0035-2

**Published:** 2014-03-14

**Authors:** Dong Deng, Chuangye Yan, Jianping Wu, Xiaojing Pan, Nieng Yan

**Affiliations:** 1 State Key Laboratory of Bio-membrane and Membrane Biotechnology, Center for Structural Biology, School of Life Sciences and School of Medicine, Tsinghua-Peking Center for Life Sciences, Beijing, 100084 China; 2 Tsinghua-Peking-NIBS Joint Program, Tsinghua University, Beijing, 100084 China

**Keywords:** TAL effectors, DNA, recognition, plasticity

## Abstract

Transcription activator-like (TAL) effectors specifically bind to double stranded (ds) DNA through a central domain of tandem repeats. Each TAL effector (TALE) repeat comprises 33–35 amino acids and recognizes one specific DNA base through a highly variable residue at a fixed position in the repeat. Structural studies have revealed the molecular basis of DNA recognition by TALE repeats. Examination of the overall structure reveals that the basic building block of TALE protein, namely a helical hairpin, is one-helix shifted from the previously defined TALE motif. Here we wish to suggest a structure-based re-demarcation of the TALE repeat which starts with the residues that bind to the DNA backbone phosphate and concludes with the base-recognition hyper-variable residue. This new numbering system is consistent with the α-solenoid superfamily to which TALE belongs, and reflects the structural integrity of TAL effectors. In addition, it confers integral number of TALE repeats that matches the number of bound DNA bases. We then present fifteen crystal structures of engineered dHax3 variants in complex with target DNA molecules, which elucidate the structural basis for the recognition of bases adenine (A) and guanine (G) by reported or uncharacterized TALE codes. Finally, we analyzed the sequence-structure correlation of the amino acid residues within a TALE repeat. The structural analyses reported here may advance the mechanistic understanding of TALE proteins and facilitate the design of TALEN with improved affinity and specificity.

**Electronic supplementary material:**

The online version of this article (doi:10.1007/s13238-014-0035-2) contains supplementary material, which is available to authorized users.

## Introduction

Transcription activator-like effector nuclease (TALEN) is becoming an important tool for genome-editing in multiple species (Bogdanove and Voytas, 2011; Huang et al., 2011; Carlson et al., 2012; McMahon et al., 2012; Streubel et al., 2012; Beumer et al., 2013; Christian et al., 2013; Doyle et al., 2013; Heigwer et al., 2013; Kim et al., 2013; Panda et al., 2013). TALENs exploit the DNA-binding domain of TAL effectors, which usually comprise 1.5 to 33.5 tandem repeats (TALE repeats), for sequence specific and customizable DNA recognition (Swarup et al., 1992; Bonas et al., 1993; Bai et al., 2000; Gu et al., 2005; White and Yang, 2009; Boch and Bonas, 2010). Each TALE repeat consists of 33 to 35, typically 34, highly conserved amino acids except for the two at positions 12 and 13, which are named RVDs for repeat variable diresidues (RVDs). RVDs are responsible for specific recognition of double-stranded (ds) DNA (Boch et al., 2009; Moscou and Bogdanove, 2009) or DNA-RNA hybrid (Yin et al., 2012). The sequence-specific RVD codes were deciphered through both experimental and bioinformatic investigations (Boch et al., 2009; Moscou and Bogdanove, 2009; Streubel et al., 2012; Yin et al., 2012; Yang et al., 2014). The well-characterized RVD codes include NI (Asn and Ile) for adenine (A), HD (His and Asp) for cytosine (C), NG (Asn and Gly) for thymine (T) and methylated cytosine (mC), NH (Asn and His) and NK (Asn and Lys) for guanine (G), NN (Asn and Asn) for G/A, and NS (Asn and Ser) for all the four bases (Boch and Bonas, 2010; Bogdanove and Voytas, 2011; Streubel et al., 2012; Yang et al., 2014).

The crystal structures of TAL effector (TALE) proteins bound to their respective target DNAs have revealed that the residue at position 13 is the only one directly involved in DNA base recognition, whereas the 12th residue stabilizes the proper loop conformation (Deng et al., 2012a; Mak et al., 2012). dHax3, an engineered TALE protein (Mahfouz et al., 2011), proved to be a useful scaffold for structural characterizations (Fig. S1A). The 1.9 Å structure of dHax3 bound to the dHax3 box DNA element elucidated the molecular basis for the recognition of bases A by Ser, C by Asp, and T by Gly (Deng et al., 2012a). In addition, the structures of dHax3 in complex with methylated DNA (Deng et al., 2012b) and DNA-RNA hybrid (Yin et al., 2012) may extend the potential application of TALE repeats. The 3.0 Å structure of another TALE protein, PthXo1, in complex with DNA has further revealed the molecular basis for the recognition of bases G by Asn and A by Ile (Mak et al., 2012).

Despite the breakthrough in the structural elucidation of DNA recognition by TALE repeats, several questions remain to be addressed. For example, compared to the simple TALE codes, Asp for C and Gly or * for T (* stands for the absence of a residue), the specificity for bases A and G appears to be more complex and perplexing. The structural basis of more TALE codes for A/G remains to be investigated. In addition, the structure-function correlation of the non-RVD residues within each TALE repeat has yet to be analyzed systematically. Here we attempt to address these questions. On top of that, we wish to propose a new demarcation system for the TALE repeat from a structural point of view given that the previously defined TALE repeat appears to be inconsistent with the basic structural unit of a TALE protein (Fig. S1A).

## Results

### Structure-based redefinition of a TALE repeat

**Figure 1:**
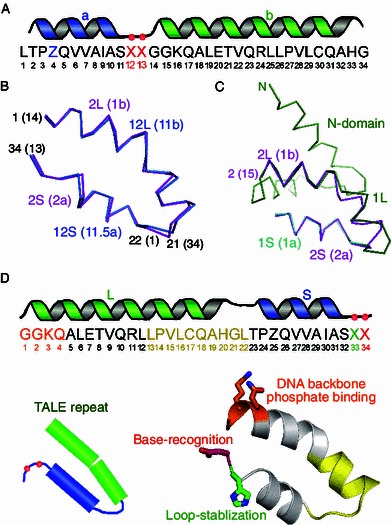
**Structure-derived redefinition of a TALE repeat**. (A) The traditional demarcation of a TALE repeat. Shown here is the secondary structure of a representative repeat in dHax3 (Deng et al., 2012a). RVD is indicated by red XX. Z stands for Glu or Gln. Please refer to Fig. S1B for the three-dimensional structure of a previously defined TALE repeat. (B) Re-demarcation of the TALE repeat allows the existence of integral number of repeats in TAL effectors. The structural elements are labeled with both new and previous (in bracket) designations. According to the new demarcation, the previous Helix a in repeat 11.5, which is identical to Helix a in any other repeat of dHax3, becomes Helix 12S of repeat 12. Similarly, the previous Helix 1b becomes Helix 2L. Within the new system, the residues in a TALE repeat are also re-numbered. The corresponding numbers within the previous system are bracketed. (C) The last helix of the N-domain in dHax3 is structurally similar to Helix b in the previously defined TALE repeat. The N-domain and the following Helix 1a (now Helix 1S) of dHax3 are colored green and cyan, respectively. Notably, the structural segment containing the last helix of the N-domain and Helix 1S can be reasonably well superimposed to the structural motif shown in Fig. 1B. Therefore, we define the last helix of the N-domain as Helix 1L of the first repeat in TAL effectors. (D) Structure-suggested re-demarcation of a TALE repeat. Based on the structures of TALE proteins, we wish to propose a new numbering system for a TALE repeat which starts with the invariant residue Gly (originally Gly14) and concludes with the base-recognition residue (originally residue 13, the 2nd residue of RVD)

According to the sequence-based definition of a TALE repeat, TAL effectors usually comprise 1.5–33.5 repeats that may recognize 2–34 DNA bases. Notably, within each repeat the two helices (Helices a and b) are positioned with an included angle of approximately 60 degrees (Figs. 1A and S1B) (Deng et al., 2012a). TAL effectors belong to the α-solenoid superfamily which also includes pentatricopeptide repeat proteins (PPR) (Yin et al., 2013), tetratricopeptide repeat proteins (TPR) (Das et al., 1998), and other proteins. The fundamental structural motif of PPR and TPR are helical hairpins. Structural scrutiny of TALE repeats in the context of the overall structure strongly suggests that the helical hairpin comprising Helix b of repeat_n_ and Helix a of repeat_n+1_ is the basic structural unit (Fig. S1A) (Deng et al., 2012a).

To better reflect the structural integrity and general structural feature of the α-solenoid superfamily, we hereby propose to re-demarcate the TALE repeat in which Helix b precedes Helix a and the base-recognition residue is placed at the last position, i.e., position 34 in most cases. The obvious advantage with this new demarcation for a TALE repeat is that any TAL effector shall end up with a complete, rather than a half, repeat (Fig. 1B), and thus shall leave only one helix to the first repeat. Notably, all the TALE repeats are preceded with a conserved N-terminal domain, and the last helix of the N-terminal domain exhibits nearly the same structural feature as Helix b in a TALE repeat despite the lack of sequence similarity (Figs. 1C and S2A). The sequence for this helical segment is almost invariant among TALE members (Fig. S2B), suggesting an identical structural feature in all TAL effectors (Deng et al., 2012a; Gao et al., 2012; Mak et al., 2012). With the N-terminal helix included, the first repeat in a TAL effector is then a helical hairpin that shares almost identical structural characteristics with all the other repeats (Fig. 1C).

With this new boundary system for a TALE repeat, the previous nomenclature of the two helices, a and b, is no longer appropriate. We propose to name Helix L and Helix S for the long and short helices within a repeat, respectively (Figs. 1D and S1C). According to the new definition of a TALE repeat, the base-recognition residue is located at the last position of a repeat instead of the previously assigned position 13 (Fig. 1D). This new numbering system has the following advantages: (1) each repeat, which now comprises two anti-parallel α-helices, is consistent with the basic structural unit of TAL effectors (Fig. S1A), and conform to the other α-solenoid superfamily proteins; (2) the number of repeats and the corresponding DNA bases can perfectly match (i.e., 12 repeats for 12 bases, repeat 1 for base 1, etc.). The TALE structures discussed hereafter in this manuscript will abide by this new numbering system.

### Structures of dHax3 variants in complex with designed DNA sequences

**Figure 2:**
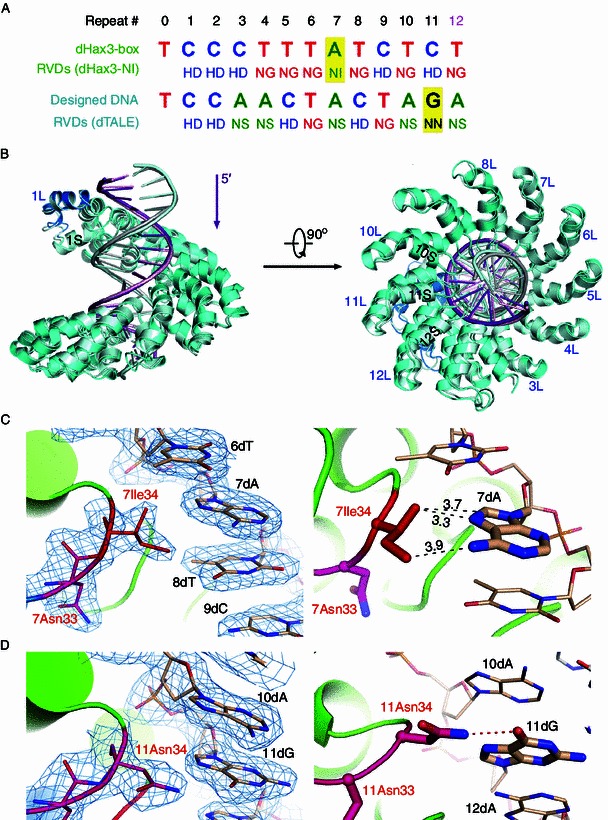
**Structures of dHax3 variants in complex with their respective target DNA elements**. (A) The sequences of the forward strand DNA and the corresponding RVDs used in the engineered dHax3 variants, which were designated dHax3-NI and dTALE, respectively. The RVDs that were not present in the reported dHax3 structure (Deng et al., 2012a) are shaded yellow. (B) Structural superimposition of DNA-bound dHax3 (grey) and dTALE (cyan). The two structures can be superimposed with an RMSD of 0.946 Å over 455 Cα atoms. The N-terminal domain of dTALE is colored blue. The PDB accession code for the DNA-bound dHax3 is 3V6T. (C and D) Structural basis for the recognition of bases A and G by Ile34 and Asn34. The 2Fo-Fc electron density map, shown in blue mesh, was contoured at 1.2 *σ*. The distances between the side group of Ile34 and base A are labeled in the unit of Å. The hydrogen bond between Asn34 and base G was indicated by red dashed line (lower right). All structure figures were prepared with PyMol (Schrodinger, 2010)

Despite the several high-resolution structures obtained for dHax3, one potential drawback of this engineered TALE protein is that it only contains limited types of TALE codes. To gain a better understanding of DNA recognition by TALE, we sought to further engineer dHax3 protein to generate more TALE codes. As a proof-of-principle, we generated two dHax3 variants, dHax3-NI, in which Ile34 was introduced at repeat 7 to replace Ser_34_ for recognition of base A (Fig. 2A), and dTALE (standing for designed TALE repeats), in which the RVDs in repeats 3–12 were replaced and the protein recognize an artificial DNA element (5′-TCCAACTACTAGA-3′) (Fig. 2A). Two structures were determined for DNA-bound dHax3-NI at 2.8 Å and 2.2 Å, respectively. The structure of dTALE in complex with the target DNA element was refined at 2.4 Å resolution (Table S1).

The structures of DNA-bound dTALE can be superimposed to dHax3 with an RMSD (root-mean-squared deviation) of 0.946 Å over 455 Cα atoms with the major deviation occurring to the amino (N) terminal helix (Fig. 2B). Similarly, structural comparison of DNA-bound dHax3 and PthXo1 reveals an RMSD of 1.04 Å over 429 Cα atoms (Fig. S3). These observations corroborated the notion that DNA binding by TALE repeats is modular and context free. Nevertheless, the new structures reveal some local variations in DNA recognition compared to that seen in the DNA-bound PthXo1.

While the coordination of base A by Ile34 is almost identical in the structures of dHax3-NI and PthXo1 (Mak et al., 2012) (Fig. 2C), the binding of base G by dTALE shows some subtle but important difference. In the PthXo1-DNA complex structure, Asn34 is hydrogen-bonded (H-bonded) to the N_7_ atom of base G; in the high-resolution structure of dTALE, however, the side group of Asn34 donates a H-bond to O_6_ atom of G (Figs. 2D and S4). Given that O_6_ atom is more electronegative than N_7_ atom in base G, the H-bond with O_6_ atom may represent a stronger interaction.

### Recognition of base A by a broad range of amino acids

**Figure 3:**
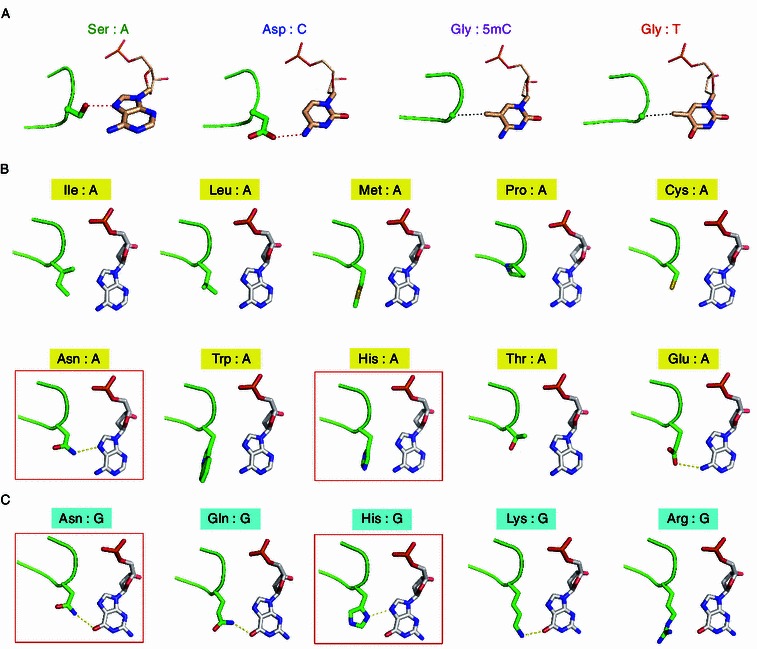
**Structural basis for the recognition of bases adenine and guanine with new TALE codes**. (A) A summary of the reported structures (Deng et al., 2012a; Deng et al., 2012b) for the recognition of the indicated DNA bases by dHax3. Shown here are the 34th residues in the TALE repeats. (B and C) Structural basis for the recognition of bases A or G by a number of predicted or unpredicted TALE codes. Each panel represents a dHax3 variant in which the 34th residue in repeat 7 was replaced with the indicated amino acid. In total fifteen structures were determined (Tables S1–3). Notably, Asn34 and His34 each bind to A and G in different ways, which provides the structural basis for the distinctive recognition strengths revealed by genetic studies (Streubel et al., 2012)

Compared to the favorable coordination of bases A by Ser34, C by Asp34, and T by Gly34 (Fig. 3A), the recognition of base A by Ile34 is rather intriguing. The hydrophobic side chain directly faces the base without favorable contact, yet the electron density map unambiguously consolidates the model building in this way (Fig. 2C). The structural observation suggests that Ile34 may not be a major contributor to the affinity of DNA binding by TALE repeats, consistent with the classification of NI as a “weak” TALE code (Streubel et al., 2012). It also insinuates that base A may “tolerate” a wide range of amino acids at position 34 of a TALE repeat when there is no steric clash. To test this speculation, we generated dHax3 variants with the 34th residue on repeat 7 substituted with different amino acids. We then launched crystallization trials for the variants in complex with dHax3 box DNA element.

In total, we obtained nine high-quality structures that elucidate the accommodation of base A by Leu, Met, Cys, Pro, Trp, His, Thr, Asn, and Glu at the 34th position (Fig. 3B, Table S2). Among these, the hydrophobic residues Leu, Met, Cys, and Pro all adopt conformations that do not clash with base A. As what happens to Ile34, there is no specific coordination found between these hydrophobic residues and base A. Trp, Thr, and His, in spite of the presence of polar groups, do not form H-bond with base A. They also appear to adopt conformations that simply avoid steric clash. These residues may all represent weak code, if they are used for base recognition. On the other hand, Asn34 and Glu34 can both be H-bonded to base A (Fig. 3B), although the recognition between Glu34 and base A was unreported previously. Interestingly, the side group of Asn34 donates an H-bond to N_7_ atom of A, reminiscent of what was seen for the coordination of base G in the DNA-bound PthXo1 structure (Fig. S4B).

### Recognition of base G by Gln, His, Lys, or Arg in the TALE repeat

When the natural TALE codes were discovered, no code was found highly specific for base G (Boch and Bonas, 2010). In light of the structures of the DNA-binding zinc finger proteins where positive charged amino acids such as Arg, His, Lys play a critical role in the coordination of base G (Fig. S5) (Pavletich and Pabo, 1991), we made a dHax3 variant with an Arg34, His34, Lys34 in the 7th repeat. In addition, given the resemblance between Asn and Gln, we generated one more dHax3 variant where the base recognition residue of repeat 7 was replaced by Gln. We then attempted to co-crystallize these proteins with a modified dHax3 box element whose 7th base is changed from A to G. Four complex structures were determined at high resolutions (Fig. 3C, Table S3).

Gln34, just as Asn34, is able to form an H-bond to the O_6_ atom of G, indicating a favorable interaction (Fig. 3C). There was no report of G-recognition by Gln34. Nevertheless, an independent study showed that Gln34 is highly specific for base G, but not for base A (Yang et al., 2014). Therefore, Gln34 may be a good code that can discriminate G from A. It was shown that His34 is a strong code for base G, but not for base A (Streubel et al., 2012; Yang et al., 2014). The structures shown here provide the molecular basis for the distinctive recognition of G and A by His34. H-bond is found between His34 and base G, but not with base A (Fig. 3B and 3C), which may explain the observation that His34 is a strong code for G, but weak for A (Streubel et al., 2012; Yang et al., 2014). Lys34 was regarded as a “weak” code for base G, although H-bond is found between the side group and the O_6_ atom of G. On the other hand, no H-bond is seen between Arg34 and base G. The long, extended side chain of Lys and Arg may make them less favorable in the context of the overall structure.

Notably, all the structures shown in Fig. 3B and 3C are obtained with one-code mutation of dHax3. It is possible that some of the residues, especially those for base A are simply tolerated here. Nevertheless, the discriminative binding of A and G by Asn34 or His34 unveiled by the structures provide unprecedented clarity to understand the biochemical observations (Streubel et al., 2012; Yang et al., 2014). Asn34 form the hydrogen bond in a different way with both A and G. The length of the hydrogen bonds are about 3 Å. Given that O_6_ atom in base G is more electronegative than N_7_ atom in base A, the H-bond with O_6_ atom may represent a stronger interaction. The new structures shown here enrich the structural gallery of the TALE codes (Fig. 3).

### Molecular basis for the structural plasticity of the TALE repeat

**Figure 4:**
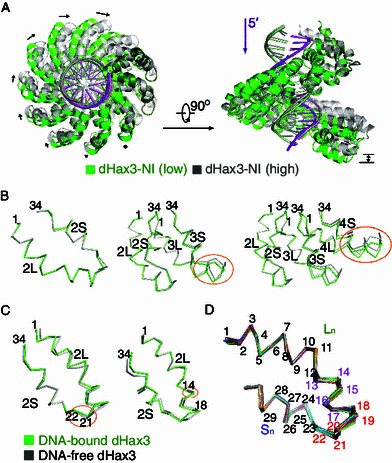
**Structural basis underlying the structural plasticity of TALE repeats**. (A) The structure of DNA-bound dHax3-NI determined at 2.2 Å (high) resolution exhibits distinct conformation from all the other DNA-bound dHax3 variants, including the same complex determined at 2.8 Å (low) resolution. The forward strands of DNA in the high and low resolution structures are colored pink and magenta, respectively. The two structures are superimposed against the 1st TALE repeat in dHax3. The N-terminal domain of the low resolution dHax3-NI is colored dark green. (B) Small structural variations in each repeat are amplified to prominent conformational changes seen in the overall structure in Fig. 4A. Shown here are the structural superimpositions against repeat 2 for one, two, and three repeats of the high (grey) and low (green) resolution structures of DNA-bound dHax3-NI. The segments that display the most pronounced structural changes are highlighted by orange circles. (C) Structural comparison of the 2nd repeat in the DNA-free (silver) and DNA-bound (green) dHax3 reveals that residues 14–22 display structural flexibility. Repeat 2 from the two structures are superimposed against either Helix S (left) or Helix L (right). (D) Structural comparison of 22 TALE repeats out of the high and low structures of dHax3-NI. For visual clarity, only Cα ribbons are shown. The residue numbers are colored from black to red with increasing deviations of the Cα atoms

During structural analysis of DNA-bound dHax3-NI, we found that while the low-resolution structure (2.8 Å) can be completely overlaid with those of dHax3 and dTALE (Fig.S3B), the high-resolution one (2.2 Å) deviates considerably from all the other DNA-bound TALE structures (Fig. 4A). Notably, the TALE repeats of dHax3 undergo pronounced conformational shifts upon DNA binding, which involves approximately 25 Å compression of the twelve TALE repeats along the helical axis of the DNA duplex, although the number of TALE repeats per superhelical turn remains unchanged (Deng et al., 2012a). In contrast, the structural difference between the high-resolution structure of DNA-bound dHax3-NI and the other DNA-bound TALE structures is a combination of both axial compression and rotational tightening of the TALE repeats accompanied by a slight distortion of the DNA duplex (Fig. 4A). The overall conformational change of the complex indicates that TALE repeats may retain structural flexibility even after binding to DNA. We then sought to identify the structural elements underlying the plasticity of TALE repeats, which may shed light on the understanding of the kinetics of DNA binding by TALE repeats.

Despite the prominent conformational changes between the overall structures, when the individual repeats of the high and low-resolution structures of DNA-bound dHax3-NI are superimposed, the RMSD value is 0.23 Å over 30 Cα atoms of the second repeat (Fig. 4B, left panel). Similar to the comparison between DNA-free and DNA-bound dHax3 structures (Fig. 4C) (Deng et al., 2012a), the overall changes result from the accumulative effect of small alterations in the φ and ϕ values of the peptide bonds of residues 14–22 (corresponding to the previously designated residues 26–34) in each repeat (Fig. 4B and 4C). Supporting this notion, the 22 repeats from both the high and low-resolution structures of dHax3-NI can be precisely superimposed with the pairwise RMSD values between 0.3–0.6 Å over 29 to 33 Cα atoms, with the deviations mainly at the segment containing residues 13–22 in each repeat (Fig. 4D). These structural observations suggest that the residues 13–22 within a TALE repeat are responsible for the structural plasticity of the repeat.

## DISCUSSION

TALENs are emerging as an important tool for genome manipulation. Despite their increasing applications, the target specificity and affinity of TALENs remain to be further improved. The advent of the crystal structures of DNA-free and DNA-bound TALE repeats provide the opportunity to further optimize TALE repeats for customized applications. The structures reported here, in combination with the previously reported structures and genetic analyses, may shed light on the selection of TALE codes for improved specificity and affinity.

Generally speaking, the recognition involving H-bond may represent stronger code, such as Asp34 for base C (Fig. 3A). Notably, the newly determined structures reported in this manuscript show that His34 forms H-bond with base G, but not with base A, which consolidates the genetic observation that His34 is a strong and specific code for base G (Streubel et al., 2012; Yang et al., 2014). The structures also suggest that Gln34 and Asn34 are both strong codes for base G. Gln34, highly specific for base G, was not reported before and should be further exploited. On the other hand, Asn34 was originally thought to be a strong code for both G and A (Fig. 3A). Recent study indicated that Asn34 favors G over A when a large number of these bases exist within a given sequence (Streubel et al., 2012; Yang et al., 2014). The structural basis for this observation is revealed (Fig. 3A and 3B).

Structures shown in Fig. 3A suggest that base A may be compatible with a broad spectrum of amino acids without specific coordination or high affinity. Similarly, Gly for base T is rather an avoidance of steric clash with the 5-methyl group of T instead of specific coordination. Therefore, when it comes to TALEN design, the codes for A and T may represent the weak codes. Asn34 is a strong code for base A only when the discrimination between A and G is not a concern with the target DNA sequences. His34/Gln34 → G and Asp34 → C can be used as strong codes. However, the *in vivo* recognition of base C may be complicated by methylation. Gly34, but not Asp34, should be applied to recognize mC (Deng et al., 2012b) (Fig. 3).

Apart from the base-recognition residue, the other residues in a TALE repeat may also contribute to binding affinity. Our structure-based definition of TALE repeat provides a more convenient way to describe the functional elements within one TALE repeat. According to this new definition, a TALE repeat of 34 amino acids contains the following functional elements: DNA backbone phosphate binding residues (BBR, residues 1–4), the residues for repeat flexibility (RF, residues 13–22), the loop stabilizing residue (LS, residue 33), and the base recognition residue (BR, residue 34). The other residues constitute the scaffold of the helices (Fig. 1D).

**Figure 5:**
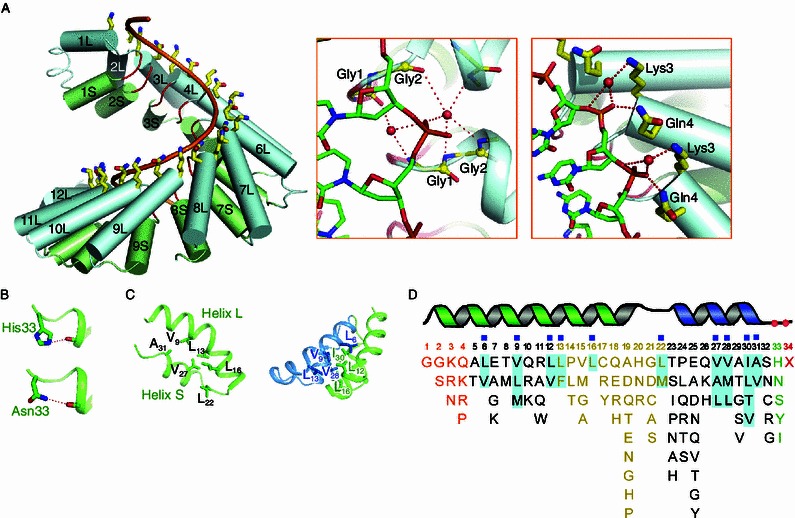
**Structural and functional analysis of the residues within a TALE repeat**. (A) The first four residues (BBR, backbone binding residues) of a TALE repeat are responsible for DNA backbone binding. Left panel: DNA-bound dHax3 with the repeats relabeled according to the new demarcation defined in this manuscript. Only the backbone of the forward strand DNA is shown. Central panel: Gly1 and Gly2 in a TALE repeat bind to DNA phosphates through water-mediated H-bonds. Right panel: Lys3 and Gln4 coordinate the backbone phosphate of the forward strand DNA through direct or water-mediated H-bonds. Water molecules are shown as red spheres. H-bonds are represented as red dashed-lines. (B) Loop-stabilizing residue, His or Asn, at position 33 provides H-bond donor to interact with the carbonyl oxygen in Helix S. (C) The intra- and inter-repeat contacts are mediated mainly through van der Waals interactions. Residues that mediate the intra-(left) and inter-(right) repeat contacts are shown as sticks. (D) Composition analysis of the residues in a TALE repeat. The residues shown here for each position is adopted from a statistics of 2023 TALE repeats (Boch and Bonas, 2010). The residues that are involved in intra- or inter-repeat contacts are indicated by the blue squares above and shaded in cyan. Note that few residues from the RF (repeat flexibility) segment, which is colored yellow, are involved in the structural stabilization

The classification of the functional elements within a TALE repeat may provide some guidelines for the design of a TALE repeat. Other than the base recognition residue discussed above, the two most prominent functional elements are BBR (residues 1–4) and LS (residue 33). The BBR motif contains amino acid sequence GGKQ in dHax3 (Fig. 1D). The first and second Gly coordinate the phosphate of sense strand DNA through water-mediated H-bonds with their backbone carbonyl oxygen and amide groups (Fig. 5A). Notably, the first Gly is invariant among all TAL effectors because any other residue with a side group at this position may cause steric clash with the DNA backbone (Fig. 5A, central panel). The second Gly may be replaced with amino acids with short side chain such as Ser or Ala. Notably, Ser was found at this position in some TAL effectors (Fig. 5D) (Boch and Bonas, 2010). Lys3 and Gln4 bind to the DNA phosphate through direct or water-mediated H-bonds (Fig. 5A, right panel). They provide the electro-positive potential to hold the negative DNA phosphate. We suggest that any residue whose side group can function as H-bond donor, such as Arg, Asn, and Thr, may work at these positions.

For LS residue at position 33, an H-bond donor is required to interact with the backbone carbonyl oxygen of Helix S (Fig. 5B). Although five amino acids (His, Asn, Ser, Tyr, Ile) were found at this position (Fig. 5D) (Boch and Bonas, 2010), His and Asn may be the ideal candidates.

Residues at certain positions within a TALE repeat are important for the intra- and inter-repeat contacts so as to preserve the structural integrity. For instance, hydrophobic residues are heavily involved in both intra- and inter-repeat interactions. They occupy positions 6, 9, 12, 13, 16, 22, 27, 28, 31, and 32 (Fig. 5C). Bioinformatic analysis of TALE repeats revealed that residues at these ten positions are almost exclusively hydrophobic and generally more conserved (Boch and Bonas, 2010) (Fig. 5D). Notably, such residues are less concentrated at the RF region. There are only three, including two that demarcate this region (residues 13 and 22) and one invariant Leu at position 16. Such arrangement may ensure the structural folding as well as the superhelical arrangement of the TALE repeats that is required for binding to the double helix. During TALEN design, residues at these positions may not be altered arbitrarily.

Apart from the functional elements and structural residues, the other positions in a TALE repeat are largely occupied by hydrophilic residues, probably to ensure the solubility of the overall protein. It is noteworthy that residues at positions 13–22 constitute the RF motif which confers the structural plasticity of TALE repeats. Hence residues within this segment may be important for the kinetics of DNA binding, and indirectly contribute to the binding affinity. Biochemical analysis and kinetic studies on DNA binding shall be applied to test TALE variants with the RF residues altered.

In sum, we report structures of fifteen dHax3 variants that reveal the structural basis of more TALE codes. We also wish to propose a new demarcation for the TALE repeat, which better reflects the structural integrity and allows a convenient description of the functional elements within a TALE repeat. Finally, upon structural analysis, we discussed some guidelines for TALEN design aiming for improved specificity and affinity.

## MATERIALS AND METHODS

### Protein preparation

All TAL effectors with residues 231–720 were subcloned into pET21b vector (Novagen). All the mutated dHax3 (231–720)s were generated by site-directed mutagenesis. To obtain purified protein for crystallization, plasmid was transformated into *E. coli* BL21 (DE3) and induced by 0.2 mmol/L isopropyl -D-thiogalactoside (IPTG) when the cell density reached an OD_600_ of 0.8. After growth at 22°C for 16 h, the cells were harvested, re-suspended in the buffer containing 25 mmol/L Tris-HCl pH 8.0, and 500 mmol/L NaCl, and disrupted using sonication. The recombinant proteins were purified sequentially through Ni^2+^-nitrilotriacetate affinity resin (Ni-NTA, Qiagen), heparin column (GE Healthcare) and desalting column (Hiprep 26/10, GE Healthcare).

### Crystallization

The forward strand DNA and the reverse strand DNA were mixed with equal molar amount, heated at 85°C for 3 min, and annealed by slow cooling to 22°C over a period of 5 h.

For crystallization, a variety of dHax3 (residues 231–720) and the 17-bp DNA duplex (for sequence, see Table 1) was mixed with a molar ratio of approximately 1:1.5 at 4°C for 30 min.

**Table 1:** The sequences of DNA duplex for crystallization

DNA1	5′-TG TCCCTTTATCTCT CT-3′
	5′-AG AGAGATAAAGGGA CA-3′
DNA2	5′-TG TCCCTTTGTCTCT CT-3′
	5′-AG AGAGACAAAGGGA CA-3′
DNA3	5′-TG TCCAACTACTAGA CT-3′
	5′-AG TCTAGTAGTTGGA CA-3′

The crystals of the protein-DNA complex were grown at 18°C by the hanging-drop vapour-diffusion method. The crystals grew to full size after 4 days in the mother solution containing 10–15% PEG3350 (*w*/*v*), 12% ethanol, and 0.1 mol/L MES pH 6.0.

The initial diffraction of the crystals was not good enough to accurately assign the side chains. For optimization, dehydration strategy was used. Briefly, the crystals were transferred into initial dehydration buffer containing mother solution plus PEG400 5% (*v*/*v*) and equilibrate in 18°C about 5 min. The crystals continue to be transferred other dehydration buffers with additional PEG400 concentration from 10% to 25% and incubated another 5 min. After dehydration, the crystals were harvested using the fiber loops and saved into the liquid nitrogen.

### Data collection and structural determination

The data sets of dHax3 variants in complex with target DNA elements were collected at SSRF (Shanghai Synchrotron Radiation Facility) beam line BL17U except the dHax3 (S505H) in complex with dHax3 box (DNA2) using CCD detector Saturn 944+ on micromax-007 HF (Rigaku). All data sets were integrated and scaled with the HKL2000 (Otwinowski and Minor, 1997). Further processing was carried out with programs from the CCP4 suite (Winn et al., 2011). The structure of DNA-protein complex were solved by molecular replacement (MR) with the reported dHax3-DNA complex structure (PDB accession code: 3V6T) as the initial searching model using the program PHASER (McCoy et al., 2007). The structure was refined with PHENIX (Adams et al., 2002) and COOT (Emsley and Cowtan, 2004) iteratively. Data collection and structural refinement statistics are summarized in Tables S1–3.

## Electronic supplementary material

Below is the link to the electronic supplementary material.

Supplementary material 1 (PDF 1561 kb)

## Abbreviations

RVDs: repeat variable diresidues
TAL: transcription activator-like
TALE: TAL effector
TALEN: transcription activator-like effector nuclease
