# Supplementary material for: Revisiting the TALE repeat
Source: Protein Cell. 2014 Mar 14;5(4):297–306. doi: 10.1007/s13238-014-0035-2 (PMC3978159; doi:10.1007/s13238-014-0035-2)
Supplement: Supplementary file 1 — Supplementary material 1 (PDF 1561 kb) [file 13238_2014_35_MOESM1_ESM.pdf]

Supplementary Figures and Legends

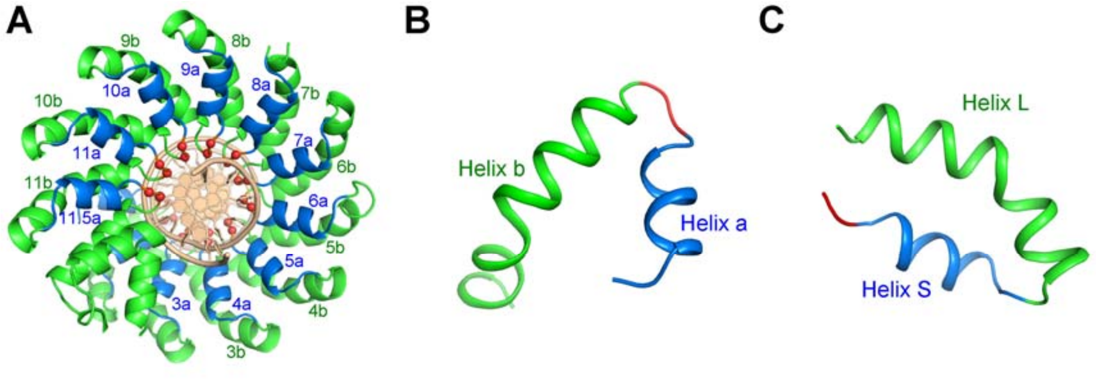

Supplementary Figure 1 | The structure of dHax3 suggests that the building block for TALE repeats is the helical hairpin. (**A**) The overall structure of dHax3 in complex with double stranded (ds) DNA. The two helices in each repeat are colored green and blue. RVDs (repeat variable di-residues) are shown as red spheres. (**B**) The previously defined TALE repeat which positions RVDs between the two helices. The two helices, Helices a and b, are positioned with an included angel of approximately 60 degree. (**C**) The structure-defined TALE repeat which positions the long helix (Helix L) preceding the short helix (Helix S). Note that this new demarcation of a TALE repeat is consistent with the fundamental structural unit seen in panel A.

A

```
dHax3-N    GGVTAVEAVHAWRNALTGAPLN
helix b  14GGKQALETVQRLLPVLCQAHG34
```

B

```
 dHax3-N   GGVTAVEAVHAWRNALTGAPLN
  Hax2-N   GGVTAVEAVHAWRNALTGAPLN
  Hax4-N   GGVTAVEAVHAWRNALTGAPLN
  TalC-N   GGVTAVEAVHASRNALTGAPLN
Pthxo6-N   GGVTAVEAVHAWRNALTGAPLN
  PthC-N   GGVTAVEAVHAWRNALTGAPLN
  PthB-N   GGVTAVEAVHAWRNALTGAPLN
  PthA-N   GGVTAVEAVHAWRNALTGAPLN
  Apl3-N   GGVTAVEAVHAWRNALTGAPLN
AvrBS3-N   GGVTAVEAVHAWRNALTGAPLN
```

Supplementary Figure 2 | Sequence alignment of the last helix of the N-terminal domain in the TALE proteins. (**A**) Sequence alignment of the last helix of the N-terminal domain of dHax3 with a representative helix b in a TALE repeat. (**B**) The sequences of the last helix of the N-terminal domain of TAL effectors are highly conserved. The sequences were aligned with ClustalW.

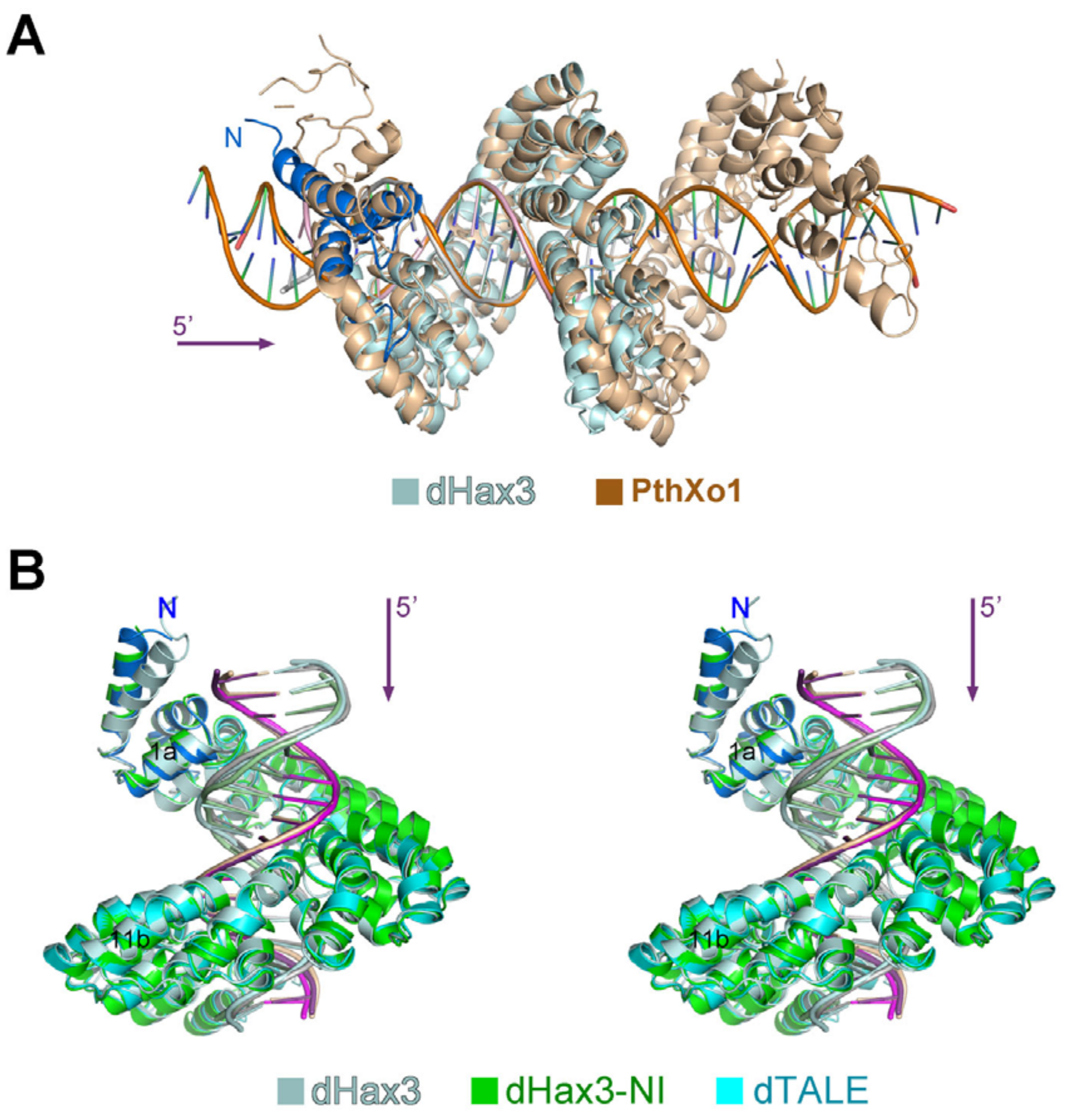

Supplementary Figure 3 | Structural comparison of different TALE proteins in complex with dsDNA. (**A**) Structural comparison of DNA-bound dHax3 (PDB code: 3V6T) and PthXo1 (PDB code: 3UGM). The two structures can be superimposed with an RMSD of 1.04 Å over 429 Cα atoms. (**B**) Structural comparison of DNA-bound dHax3 variants. Shown here is the low resolution structure of dHax3-NI. Please refer to Figure 2A for the nomenclature of the dHax3 variants.

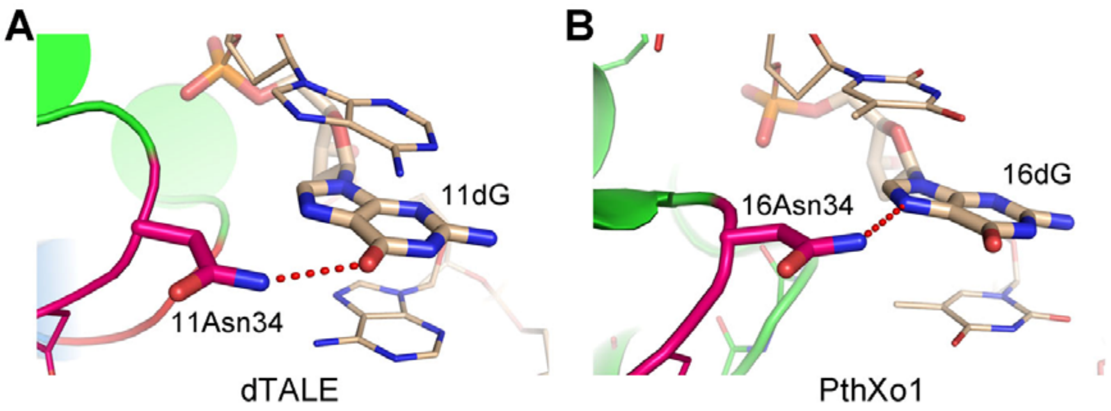

Supplementary Figure 4 | Difference in the coordination of base G by Asn_{34} shown in structures of dTALE and PthXo1. The structures of DNA-bound dTALE and PthXo1 were determined at 2.4 Å and 3.0 Å, respectively.

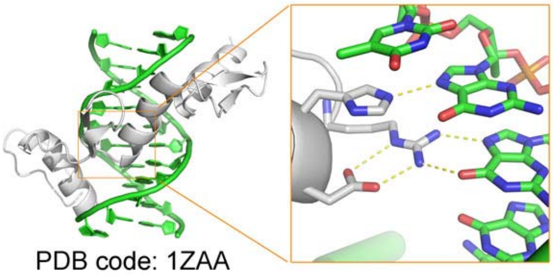

Supplementary Figure 5 | A representative structure of the DNA base recognition by zinc finger proteins.

Table S1. Data collection and refinement statistics

	dTALE	dHax3-NI (low)	dHax3-NI (high)
**Data collection**			
Space Group	$P2_1$	$P2_1$	$P2_1$
Cell dimensions			
a, b, c (Å)	84.63, 88.13, 90.48	84.29, 89.36, 90.27	84.53, 80.99,89.08
α, β, γ, (°)	90, 104.96, 90	90, 103.74, 90	90, 103.62, 90
Number of molecules in ASU	2	2	2
Wavelength (Å)	0.9792	0.9792	1.0092
Resolution (Å)	40~2.40 (2.49~2.40)	40~2.80 (2.90~2.80)	40~2.20 (2.28~2.20)
R_{merge} (%)	6.6 (47.5)	6.5 (82.2)	10.2(58.8)
I / σI	28.4(3.4)	28.9 (2.2)	19.0 (2.4)
Completeness (%)	99.7 (100.0)	99.7 (99.8)	98.6 (90.5)
Redundancy	3.8(3.8)	4.1 (4.2)	3.6 (3.3)
Wilson B factor ($Å^2$)	38.9	67.5	27.0
**Refinement**			
Resolution (Å)	40~2.40	40~2.80	40~2.20
No. reflections	50,310	32,103	58,949
R_{work} / R_{free} (%)	21.44/25.51	24.28/28.64	20.49/24.60
No. atoms			
Protein	7112	7015	7037
Main chain	3928	3880	3908
Side chain	3184	3135	3129
DNA	1362	1342	1246
Water	260	39	282
B-factors			
Protein	54.12	88.82	44.00
Main Chain	53.34	88.22	42.72
Side Chain	55.09	89.56	45.61
DNA	53.42	79.85	41.61
Water	49.93	77.06	41.01
R.m.s. deviations			
Bond lengths (Å)	0.009	0.010	0.009
Bond angles (°)	1.191	1.471	1.362
Ramachandran plot statistics (%)			
Most favoured	90.4	81.1	93.3
Additional allowed	9.6	18.7	6.7
Generously allowed	0.0	0.2	0.0
Disallowed	0.0	0.0	0.0

One crystal was used for each structure.

Values in parentheses are for the highest resolution shell. $R_{merge}=\Sigma_h\Sigma_i|I_{h,i}-I_h|/\Sigma_h\Sigma_i I_{h,i}$, where I_h is the mean intensity of the *i* observations of symmetry related reflections of *h*. $R=\Sigma|F_{obs}-F_{calc}|/\Sigma F_{obs}$, where F_{calc} is the calculated protein structure factor from the atomic model (R_{free} was calculated with 5% of the reflections selected randomly).

Table S2. Data collection and refinement statistics

	**Leu:A**	**Met:A**	**Pro:A**	**Cys:A**	**Trp:A**
**Data collection**					
Space Group	$P2_1$	$P2_1$	$P2_1$	$P2_1$	$P2_1$
Cell dimensions					
a, b, c (Å)	81.15, 87.38,88.25	81.00, 87.56,88.00	81.31,86.28, 86.21	80.81, 86.99, 87.64	81.44, 87.07,87.97
α, β, γ, (°)	90, 102.96, 90	90, 102.96, 90	90,102.42,90	90.00,102.81, 90.00	90, 103.24, 90
Number of molecules in ASU	2	2	2	2	2
Wavelength (Å)	0.9795	0.9795	0.9795	0.9793	0.9793
Resolution (Å)	50~1.95 (2.02~1.95)	50~2.00 (2.07~2.00)	50~2.60 (2.69~2.60)	40~2.20 (2.28~2.20)	40~2.70 (2.80~2.70)
R_{merge} (%)	9.0(80.1)	8.3(67.8)	13.4(68.8)	6.9(54.6)	9.6(50.6)
I/σ	14.9(2.0)	17.1(2.6)	11.3(2.3)	19.6(2.5)	14.8(2.6)
Completeness (%)	99.9(100.0)	99.9(99.8)	99.9(99.7)	99.8(99.4)	98.7(99.5)
Redundancy	3.8(3.8)	3.7(3.7)	3.7(3.6)	3.7(3.6)	3.2(3.2)
Wilson B factor (Å^2)	27.1	27.7	57.2	34.5	58.8
**Refinement**					
Resolution (Å)	50~1.95	50~2.00	50~2.60	40~2.20	40~2.70
No. reflections	88109	81269	35135	80636	32872
R_{work} / R_{free} (%)	19.20/22.50	20.00/23.66	21.74/27.45	21.17/24.39	22.03/26.73
No. atoms					
Protein	7233	7178	7178	7197	7143
Main chain	3992	3964	3948	3976	3960
Side chain	3241	3214	3195	3221	3218
DNA	1344	1381	1381	1362	1344
Water	692	596	114	202	63
B-factors					
Protein	34.3	39.5	46.0	44.8	50.0
Main Chain	33.1	38.5	45.6	44.0	49.3
Side Chain	35.7	40.8	46.5	45.9	50.8
DNA	39.8	45.1	47.9	51.1	54.4
Water	39.0	41.1	38.7	46.1	42.7
R.m.s. deviations					
Bond lengths (Å)	0.009	0.009	0.009	0.009	0.009
Bond angles (°)	1.333	1.340	1.444	1.338	1.363
Ramachandran plot statistics (%)					
Most favoured	93.5	93.2	89.3	93.7	91.3
Additional allowed	6.3	6.4	10.6	6.0	8.5
Generously allowed	0.1	0.4	0.1	0.2	0.2
Disallowed	0	0	0	0	0

	Thr:A	His:A	Asn:A	Glu:A
**Data collection**				
Space Group	$P2_1$	$P2_1$	$P2_1$	$P2_1$
Cell dimensions				
a, b, c (Å)	81.10, 87.02, 87.79	81.19, 87.45, 87.97	84.41, 80.81, 88.82	81.95, 87.65, 87.44
α, β, γ, (°)	90.00, 103.00, 90	90.00,102.70, 90.00	90, 103.67, 90	90.00, 102.55, 90
Number of molecules in ASU	2	2	2	2
Wavelength (Å)	0.9792	0.9785	0.9793	0.9795
Resolution (Å)	50~2.50 (2.59~2.50)	50~2.60 (2.69~2.60)	40~2.80 (2.90~2.80)	50~2.30 (2.38~2.30)
R_{merge} (%)	10.2(48.2)	11.8(65.6)	12.2 (40.0)	8.2(59.8)
I/σ	14.0(2.7)	15.4(3.5)	12.4 (2.1)	16.7(2.4)
Completeness (%)	99.6(99.0)	100.0(99.9)	98.3 (91.1)	99.9(100.0)
Redundancy	3.6(3.3)	3.8(3.7)	3.5 (2.1)	3.7(3.7)
Wilson B factor ($Å^2$)	49.8	46.8	38.7	41.4
**Refinement**				
Resolution (Å)	50~2.50	50~2.60	40~2.80	50~2.30
No. reflections	41335	44006	28,507	53514
R_{work} / R_{free} (%)	20.20/25.42	21.50/25.73	21.36/ 27.09	20.22/24.21
No. atoms				
Protein	7214	7203	7046	7178
Main chain	3980	3977	3904	3948
Side chain	3234	3226	3142	3198
DNA	1344	1381	1305	1383
Water	181	255	39	319
B-factors				
Protein	38.4	33.2	54.47	47.6
Main Chain	37.7	33.0	54.18	46.9
Side Chain	39.2	33.5	54.83	48.4
DNA	44.9	35.0	57.45	49.3
Water	34.1	28.9	44.77	44.6
R.m.s. deviations				
Bond lengths (Å)	0.009	0.009	0.009	0.010
Bond angles (°)	1.334	1.346	1.416	1.421
Ramachandran plot statistics (%)				
Most favoured	91.9	92.1	89.2	92.5
Additional allowed	8.1	7.6	10.6	7.5
Generously allowed	0	0.4	0.2	0
Disallowed	0	0	0.0	0

One crystal was used for each structure.

Table S3. Data collection and refinement statistics

	His:G	Gln:G	Lys:G	Arg:G
**Data collection**				
Space Group	$P2_1$	$P2_1$	$P2_1$	$P2_1$
Cell dimensions				
a, b, c (Å)	81.53, 87.61, 87.84	81.08, 87.07, 87.95	81.05, 87.37, 88.10	81.76,87.58, 88.15
α, β, γ, (°)	90.00,102.72, 90.00	90.00, 103, 90	90, 102.94, 90	90,102.99,90
Number of molecules in ASU	2	2	2	2
Wavelength (Å)	1.5418	0.9793	0.9795	0.9795
Resolution (Å)	40~2.45 (2.54~2.45)	40~2.40 (2.49~2.40)	50~1.95 (2.02~1.95)	50~2.25 (2.33~2.25)
R_{merge} (%)	7.5 (32.3)	10.6(78.7)	8.8(72.2)	12.3(61.7)
I/σ	22.7(4.0)	13.4(1.8)	16.0(2.1)	10.6(2.3)
Completeness (%)	96.2 (93.1)	99.9(99.6)	99.9(100.0)	95.7(97.1)
Redundancy	3.6 (2.8)	3.7(3.7)	3.8(3.8)	3.2(3.1)
Wilson B factor (Å^2)	48.2	46.9	25.4	35.8
**Refinement**				
Resolution (Å)	40~2.45	40~2.40	50~1.95	50~2.25
No. reflections	42796	46753	87813	54183
R_{work} / R_{free} (%)	22.48/25.25	21.50/25.04	20.35/23.66	20.30/24.85
No. atoms				
Protein	7161	7204	7207	7184
Main chain	3952	3972	3972	3972
Side chain	3209	3232	3235	3212
DNA	1383	1344	1346	1384
Water	403	119	689	386
B-factors				
Protein	37.5	47.4	34.8	36.4
Main Chain	36.8	46.7	34.1	35.6
Side Chain	38.2	48.3	35.6	37.4
DNA	38.2	52.9	38.0	39.0
Water	34.8	52.0	39.1	34.3
R.m.s. deviations				
Bond lengths (Å)	0.009	0.009	0.009	0.009
Bond angles (°)	1.385	1.325	1.335	1.351
Ramachandran plot statistics (%)				
Most favoured	91.0	92.6	93.7	92.4
Additional allowed	9.0	7.2	6.1	7.6
Generously allowed	0	0.1	0.1	0
Disallowed	0	0	0	0

One crystal was used for each structure.
